# The effects of elevated temperature and ocean acidification on the metabolic pathways of notothenioid fish

**DOI:** 10.1093/conphys/cox019

**Published:** 2017-03-24

**Authors:** Laura A. Enzor, Evan M. Hunter, Sean P. Place

**Affiliations:** 1 United States Environmental Protection Agency, Gulf Ecology Division, Gulf Breeze, FL 32561, USA; 2 Department of Biological Sciences, University of South Carolina, Columbia, SC29208, USA; 3 Department of Biology, Sonoma State University, Rohnert Park, CA94928, USA

**Keywords:** Energetics, global climate change, metabolism, notothenioid, ocean acidification, thermal stress

## Abstract

The adaptations used by notothenioid fish to combat extreme cold may have left these fish poorly poised to deal with a changing environment. As such, the expected environmental perturbations brought on by global climate change have the potential to significantly affect the energetic demands and subsequent cellular processes necessary for survival. Despite recent lines of evidence demonstrating that notothenioid fish retain the ability to acclimate to elevated temperatures, the underlying mechanisms responsible for temperature acclimation in these fish remain largely unknown. Furthermore, little information exists on the capacity of Antarctic fish to respond to changes in multiple environmental variables. We have examined the effects of increased temperature and *p*CO_2_ on the rate of oxygen consumption in three notothenioid species, *Trematomus bernacchii*, *Pagothenia borchgrevinki*, and *Trematomus newnesi*. We combined these measurements with analysis of changes in aerobic and anaerobic capacity, lipid reserves, fish condition, and growth rates to gain insight into the metabolic cost associated with acclimation to this dual stress. Our findings indicated that temperature is the major driver of the metabolic responses observed in these fish and that increased *p*CO_2_ plays a small, contributing role to the energetic costs of the acclimation response. All three species displayed varying levels of energetic compensation in response to the combination of elevated temperature and *p*CO_2_. While *P. borchgrevinki* showed nearly complete compensation of whole animal oxygen consumption rates and aerobic capacity, *T. newnesi* and *T. bernacchii* displayed only partial compensation in these metrics, suggesting that at least some notothenioids may require physiological trade-offs to fully offset the energetic costs of long-term acclimation to climate change related stressors.

## Teaser

Organisms inhabiting the sub-zero waters surrounding Antarctica display remarkably narrow tolerances for environmental change. This study assessed three closely related fish exposed to simultaneous changes in oceanic conditions to ascertain the impact additive stress has on their capacity to acclimate and whether or not these fish employ similar metabolic responses.

## Introduction

Anthropogenic CO_2_ emissions are having profound impacts on the chemistry of the world's oceans. It is estimated that 25–30% of the emitted carbon dioxide is absorbed by the worlds’ oceans (Ciais *et al*., 2013), resulting in a fundamental shift in the balance of carbonate species, and freeing up protons, which in turn decreases the pH of seawater in a process termed ‘ocean acidification’ (Hughes, 2000; Fabry *et al*., 2008).

While these changes in ocean chemistry are expected to impact most marine biota to some degree, regional differences in environmental conditions will likely alter the magnitude and/or rate at which these impacts on marine biota are experienced. For example, increased global *p*CO_2_ averages associated with ocean acidification (OA) may exacerbate transient spikes in *p*CO_2_ levels associated with upwelling in coastal oceans (Hales *et al*., 2005; Hauri *et al*., 2009; Thomsen *et al*., 2010; Gruber, 2011) or more rapidly accumulate in polar oceans where the extreme cold waters absorb greater amounts of CO_2_. Indeed, it is postulated that the Southern Ocean will reach a state of under saturation of aragonite as soon as the year 2030 (McNeil and Matear, 2008; McNeil *et al*., 2010). Furthermore, OA is expected to act in combination with other naturally occurring environmental stressors (e.g. hypoxia, salinity) and will in fact change concomitantly with other anthropogenically driven stressors such as rising sea surface temperatures (SST). Subsequently, it becomes important to investigate the physiological and biochemical pathways challenged by the effects of climate change on marine organisms in order to understand whole organism and subsequent ecosystem level consequences. This is especially true for organisms that are likely to experience these impacts more rapidly, such as marine organisms inhabiting the polar regions.

Ocean acidification has recently been shown to present numerous challenges for fish, including issues of re-establishing acid–base balance, olfaction impairment and predator avoidance (see Heuer and Grosell, 2014 for a review). While the effects of OA have become a well-studied topic over the past decade, we still have little insight into the capacity of organisms to respond to the interaction between ocean acidification and other environmental stressors. These interactions are key to determining how organisms will potentially respond to environmental change as perturbations occur concomitantly (Todgham and Stillman, 2013). In particular, the energy budget of an organism can be heavily impacted by the amount and type of stress the organism undergoes (Sokolova, 2013), having long-term effects on growth, reproduction, and eventually population numbers.

The dominant fish fauna of the Southern Ocean, the notothenioids, have evolved in arguably the coldest and most oceanographically stable environment found on the planet and display a remarkably narrow thermal window in which they can maintain physiological function (Somero and DeVries, 1967; Podrabsky and Somero, 2006). Given the narrow thermal window in which the notothenioids exist, it can be predicted that these stenotherms may display a significant decrease in physiological performance when confronted with an increase in temperature and/or *p*CO_2_. Previous studies on polar fishes have focused on temperature as a stress alone (Davison *et al*., 1990; Seebacher *et al*., 2005; Pörtner, 2008; Robinson and Davison, 2008a, b), or if the effect of multiple stressors were examined, a focus was placed on aerobic metabolism, leaving anaerobic pathways largely unexplored (Strobel *et al*., 2012, 2013a, b; Enzor *et al*., 2013; Magnoni *et al*., 2013; Martinez *et al*., 2013). To this end, we set out to determine the energetic response and the possible use of anaerobic pathways as compensation in three species of notothenioid fish, *Trematomus bernacchii* (Boulenger, 1902), *Pagothenia borchgrevinki* (Boulenger, 1902), and *Trematomus newnesi* (Boulenger, 1902) to an increase in temperature and *p*CO_2_. In addition to gathering routine metabolic rates (RMR's), we calculated estimates of Fulton's Index (Fulton, 1902; Peig and Green, 2010) and fish growth rates, in an effort to determine if fish maintained strong growth potential over the course of the experiment. These whole animal metrics were combined with tissue level biochemical analyses to gain insight into the underlying processes that may manifest in observed changes at the level of the whole organism.

As gills are directly exposed to the outer aquatic environment, they have multiple functions including gas exchange, acid–base balance and ionic/osmotic regulation that are likely to be directly affected by ocean acidification (Evans *et al*., 2005). These processes are energetically expensive (Hirose *et al*., 2003; Evans *et al*., 2005) with estimates of the metabolic costs of gill function being ~7% of whole animal oxygen consumption level (Mommsen, 1984) which can lead to detectable changes in whole organism metabolic rates when these functions are perturbed by environmental variation.

Furthermore, previous works on the bioenergetics of osmoregulation suggests a major source of glycolytic substrate used in gill tissues comes from glycogen stores in both the gill and liver (Perry and Walsh, 1989; Soengas *et al*., 1991; Morgan *et al*., 1997; Chang *et al*. 2007). Notably, hepatic metabolism was found to play a role in energetic compensation to salinity changes in euryhaline fishes (Nakano *et al*., 1998; Sangiao-Alvarellos *et al*., 2003), suggesting an energetic link between gill and liver organs during acclimation to environmental perturbations. As such, in addition to the whole animal metrics, we also monitored changes in metabolic capacity in gill and liver tissues isolated from each species by measuring the activity of citrate synthase and lactate dehydrogenase, biochemical markers for aerobic and anaerobic metabolism, respectively (Cai and Adelman, 1990; Hochachka and Somero, 2002). Lastly, given the reliance on lipid substrate for energy production in notothenioid fishes (Lin *et al*., 1974; Clarke *et al*., 1984), we measured total triglyceride content of liver and white muscle to determine if changes in metabolic capacity resulted in a depletion of energy stores in these tissues that represent major lipid reservoirs in these fish (Eastman, 1993).

## Materials and methods

### Seawater manipulation

We used an experimental *p*CO_2_ manipulation system first described by Fangue *et al*. (2010; adapted for large-scale use) combined with thermostated titanium heaters to create our four experimental treatments. Briefly, atmospheric air was pumped through columns filled with Drierite to remove moisture, and then columns filled with Sodasorb to scrub air of CO_2_. CO_2_-free air was then blended with pure CO_2_ using digital mass-flow controllers in order to create desired *p*CO_2_ levels. Blended air was then bubbled into flow-through header tanks, which in turn supplied experimental tanks with CO_2_-infused water via Venturi injectors. Daily measurements of salinity (using a YSI 3100 Conductivity meter, Yellow Springs, OH, USA) and temperature (using a calibrated digital thermocouple, Omega Engineering Inc., Stamford, CT, USA) were taken from all experimental tanks as well as incoming seawater. We followed standard operating procedures from the Best Practices Guide (Riebesell *et al*., 2010) for daily spectrophotometric pH measurement (total scale) using m-cresol purple and total alkalinity measurement using open-celled titration (measured using a T50 Titrator, Mettler Toledo, Columbus, OH, USA). These measurements were combined with temperature and salinity data and input into the program CO2Calc (Robbins *et al*., 2010) to calculate all other carbonate parameters. Mean values of temperature and *p*CO_2_ level ± SE are reported in Table 1.

**Table 1:** Mean measurements of *p*CO_2_ and temperature ± SE over the course of the 2011 and 2012 field seasons

2011 Season	*p*CO_2_ (µatm)	Temperature (°C)	2012 Season	*p*CO_2_ (µatm)	Temperature (°C)
Incoming seawater	417.15 ± 12.26	−1.24 ± 0.08	Incoming seawater	427.66 ± 23.97	−1.03 ± 0.152
Low temperature + low *p*CO_2_	438.82 ± 16.08	−0.61 ± 0.17	Low temperature + low *p*CO_2_	432.04 ± 22.50	−0.707 ± 0.153
Low temperature + high *p*CO_2_	953.89 ± 50.38	−0.45 ± 0.16	Low temperature + high *p*CO_2_	1024.76 ± 94.20	−0.578 ± 0.150
High temperature + low *p*CO_2_	525.11 ± 21.07	4.02 ± 0.44	High temperature + low *p*CO_2_	525.16 ± 22.41	3.86 ± 0.484
High temperature + high *p*CO_2_	1026.66 ± 9.03	4.22 ± 0.56	High temperature + high *p*CO_2_	1053.44 ± 71.87	4.03 ± 0.316

### Fish collection and experimental design

*Trematomus bernacchii, T. newnesi* and *P. borchgrevinki* were collected from McMurdo Sound, Antarctica using hook and line through 10-inch holes drilled in the sea ice. Fish were collected from October through December 2011 and September through December 2012. Once collected, fish were transported back to McMurdo Station in aerated coolers where they were acclimated for one week in a flow-through aquarium (2400-L) in ambient seawater (−1.5°C and ~430 µatm CO_2_).

After the initial acclimation period, fish were randomly placed into one of four flow-through experimental treatment tanks (1240-L each) in order to assess the response to increased temperature (4°C; a temperature these species are known to tolerate for longer periods of time; Somero and DeVries, 1967; Gonzalez-Cabrera *et al*., 1995) increased *p*CO_2_ (1000 µatm, IPCC A1F1 scenario) or a combination of increased temperature and increased *p*CO_2_. The treatment tanks consisted of a tank held at ambient conditions (control treatment; −1°C and 430 µatm), a low temperature + high *p*CO_2_ tank (−1°C and 1000 µatm), a high temperature + low *p*CO_2_ tank (4°C and 430 µatm), and a high temperature + high *p*CO_2_ tank (4°C and 1000 µatm). Fish were acclimated to experimental treatments for a period of *t* = 7, 28, 42 and 56 days (*T. bernacchii* and *P. borchgrevinki* only). Unfortunately, due to logistical constraints imposed by the relatively short field season in the Antarctic, we were unable to obtain a 56-day acclimation time point for *T. newnesi*. Experiments were replicated over the course of two field seasons to collect sufficient numbers of fish at each acclimation time point for tissue-level analyses and data from both field seasons were combined. To reduce the potential for tank effects, treatments were alternated among tanks between field seasons.

While in experimental treatment tanks, fish were fed frozen anchovy to satiation once every 3 days. After a 24h period, all remaining food was removed from the tank to prevent the build-up of waste products. Experimental tanks were sampled daily for the presence of ammonia, nitrites and nitrates; no discernible levels were detected (data not shown). At each experimental endpoint fish were removed, anesthetized in MS-222, and sacrificed by spinal transection. Liver and gill tissues were collected and flash-frozen in liquid nitrogen, and then transported back to our home institution on dry ice where they were housed at −80°C until used. All fish used in this study were housed and sacrificed according to approved animal use protocols dictated by the Institutional Animal Care and Use Committee at the University of South Carolina (USC IACUC protocol # 2018-100377-071511).

### Whole animal energetics

#### Fulton's index and specific growth rate

In order to determine the acclimation effects to the various treatments on fish condition and growth, we gathered standard length and weight measurements over the course of the experiment for *n* = 10 fish per species, per treatment at *t* = 0, 7, 28 and 42 days and *n* = 5 fish per treatment at *t* = 56 days for *T. bernacchii* and *P. borchgrevinki* unless otherwise stated (see Table 2 and supplemental data, S1 & S2).

**Table 2:** Fish condition, growth parameters and lipid concentrations for *Trematomus bernacchii*

	Acclimation time (d)	Low temp + Low *p*CO_2_	Low temp + High *p*CO_2_	High temp + Low *p*CO_2_	High temp + High *p*CO_2_
Mortality (sample size)	7				
	28				
	42			1 (day 35) (*n* = 9)	2 (day 34 & 40) (*n* = 8)
	56				
*k*	T0	1.434 ± 0.04	1.414 ± 0.04	1.416 ± 0.04 ^a^	1.431 ± 0.03^a^
	7	1.402 ± 0.05	1.393 ± 0.05	1.308 ± 0.03 ^b^	1.332 ± 0.05 ^b^
	28	1.394 ± 0.06	1.416 ± 0.04	1.340 ± 0.04 ^b^	1.344 ± 0.03 ^b^
	42	1.402 ± 0.04	1.416 ± 0.04	1.354 ± 0.03 ^b^	1.320 ± 0.04 ^b^
	56	1.395 ± 0.04	1.444 ± 0.05	1.273 ± 0.03^b^	1.341 ± 0.04 ^b^
SGR (% M day ^−1^)	7	−0.095 ± 0.13^a^	−0.369 ± 0.11^b^	−0.429 ± 0.19^b^	−0.429 ± 0.09^b^
	28	−0.037 ± 0.05^a^	−0.162 ± 0.09^a,b^	−0.229 ± 0.03^b^	−0.212 ± 0.03^b^
	42	−0.081 ± 0.04^a^	−0.081 ± 0.04^a^	−0.150 ± 0.04^a^	−0.095 ± 0.05^a^
	56	−0.081 ± 0.02^a^	−0.130 ± 0.04^a,b^	−0.155 ± 0.02^b^	−0.154 ± 0.01^b^
Liver lipids (*n* = 9)	7	4.344 ± 1.12	3.483 ± 0.60	4.883 ± 0.77	4.330 ± 0.42
	28	4.414 ± 0.67	4.468 ± 0.81	4.947 ± 0.52	5.397 ± 0.62
	56	3.768 ± 0.18	4.989 ± 1.03	5.436 ± 0.44	5.506 ± 0.64
WM lipids (*n* = 9)	7	0.626 ± 0.08	0.238 ± 0.10	0.079 ± 0.10	0.626 ± 0.05
	28	0.606 ± 0.11	0.313 ± 0.13	0.104 ± 0.20	0.606 ± 0.10
	56	0.813 ± 0.02	0.328 ± 0.13	0.116 ± 0.27	0.813 ± 0.08

Data are means ± SE; number of fish (*n*) = 10 for all treatments except 56 day time points (*n* = 5), unless otherwise stated. Fulton's condition index (*k*), superscript letters denote significant differences between time points within a treatment. Specific growth rate (SGR, % change in mass (M) per day ± SE), superscript letters denote significant differences between treatments. Lipid content (total triglycerides gfw^−1^, ± SE) of liver and white muscle (WM) of *T. bernacchii* at each experimental time point.

Fulton's Indices were calculated from the following equation:

$$K=\frac{100,000\;*\;W}{L^{3}}$$

Where *W*  is the weight in grams and *L* the standard length in mm

Specific growth rates (%d^−1^) were calculated using the equation:

$$\mathrm{SGR}=\frac{({\ln\;W}_{t}-{\ln\;W}_{i})\;*\;100}{t}$$

Where *W*_t_ is the weight at time point (g), *W*_i_  the initial weight at day 0 (g) and *t*  the experimental acclimation time in days (Hopkins, 1991).

#### Routine metabolic rates

In the 2012 season, a sub-set of fish (*n* = 5 per species) acclimated in the same treatment tanks as other fish, were evaluated for oxygen consumption rates at each experimental endpoint. Unlike the RMR's reported in a previous study (Enzor *et al*., 2013) we utilized a repeated measures design to reduce within treatment variation and reduce the total number of fish needed for this portion of the study. Our previous work suggested some notothenioid fishes display incomplete RMR compensation over a 28-day acclimation period (Enzor *et al*., 2013). To determine if these fish were capable of fully compensating when given an extended acclimation period, we also extended the acclimation period in 2012 out to 56 days.

Repeated measures of RMR for *n* = 5 fish per species, per treatment were determined over the course of the experimental acclimation period using an automated intermittent respirometry system (Loligo Systems, Denmark). Respirometry chambers were housed in covered 99-L tanks, which received a continuous flow of seawater from their respective treatment tanks. All tanks were partially submerged within an 850-L seawater table with a continuous flow of ambient seawater in order to maintain the low temperature of the cold-water treatments. Tanks which were used with warm-acclimated fish were then fitted with titanium aquarium heaters to maintain 4°C water consistently. As a precaution against confounding effects of circadian rhythms, all respirometry measurements were recorded at the same time of day (between 8:00 p.m. and 8:00 a.m.) when human activities in the aquarium space were minimal. Before any fish were placed in respirometry chambers, empty chambers were run to ensure bacterial respiration was minimal in the system (no significant respiration measured, data not shown).

After acclimation in the experimental tanks for *t*= 7, 28, 42 and 56 days (*T. bernacchii* and *P. borchgrevinki* only), fish which had been fasted for at least 48 h were placed in respirometry chambers with the flush pumps running, air bubbles were removed and chambers were then sealed. Fish were acclimated to the respirometry chamber for 2 h prior to initiating the ${\overset{˙}{M}O}_{2}$ measurements. Over the course of 10 h, oxygen consumption rates were monitored using repeated cycles consisting of a 20 min measurement period followed by a 5 min flush period to re-oxygenate the chamber. Mean ${\overset{˙}{M}O}_{2}$ values were calculated by averaging five sequential measurements whose values had an *R*^2^ value > 0.95 for the slope describing the rate of oxygen consumption after ${\overset{˙}{M}O}_{2}$ values had stabilized, typically ~6–8 h after measurement began (see supplemental information, S3). Oxygen consumption rates were standardized to a 100-g fish (Steffensen, 2005) using a mass-exponent of −0.25 (Schmidt-Nielsen, 1984). Following determination of RMR, the fish were returned to the experimental tanks and allowed to continue acclimating to the experimental conditions.

Analysis of the slopes of the respiration rate curves demonstrated ${\overset{˙}{M}O}_{2}$ values were still marginally declining after the 12h acclimation/measurement period (*m* = −0.0124 ± 0.0015, see supplemental information, S3). As such, these fish likely need to be acclimated to the respirometry chambers for longer periods to achieve more representative baseline RMR levels. As we observed no significant differences between the slopes of fish from different treatments, this likely had little to no impact on the treatment effects observed in these fish.

### Biochemical analyses

Sample size for each biochemical analysis described below was *n* = 9 fish per species, per treatment at *t* = 0, 7, 28 and 42 days and *n* = 5 fish per treatment at *t*= 56 days for *T. bernacchii* and *P. borchgrevinki* unless otherwise stated (see Table 2 and supplemental data, S1 & S2).

#### Total triglycerides

Infinity™ Triglycerides Reagent was used in order to quantify the total triglycerides present in liver and white muscle tissues to capture changes in lipid levels after short and intermediate acclimation times as well as the experimental endpoint for each species. Approximately 50mg of tissue was homogenized in ice-cold 1× phosphate buffer solution with 1% Triton-X. A standard curve was run with each plate using a Stan-Bio Triglyceride Standard (2g/L). All samples and standards were kept on ice and run in duplicate, with wells filled with deionized (DI) water as a blank. Six microliter of each standard was combined with 294 µL of Infinity Reagent, and 10 µL of each sample was combined with 290 µL of Infinity Reagent. Plates were incubated in the dark on an orbital shaker, after which they were immediately read at 500 nm on a 96-well plate reader (Bio-Tek) using pathway correction. Total triglycerides were calculated using the equation describing the slope of the standard curve (*R*^2^ value > 0.95) and were reported as total triglyceride per gram fresh tissue weight.

#### Citrate synthase activity

We used a spectrophotometric method to quantify total citrate synthase (CS) activity in both liver and gill tissues. Approximately 20 mg of tissue was homogenized on ice in a 50 mM potassium phosphate buffer (pH = 6.8). Once extracted, supernatant was stored at −20°C until ready for use.

All samples were run in duplicate and enzyme activity was measured at −1°C (maintained by glycol/water jacketed, temperature-controlled cells within the spectrophotometer), at 412 nm, over a period of 5 min. A reference cuvette was placed in the spectrophotometer (Shimadzu 1800 UV/Vis) with ~2.0 mL of CS cocktail (50 mM Imidazole-HCl, pH = 8.2, 15 mM MgCl_2_, 0.8 mg/mL DTNB, 3 mg Acetyl CoA) to measure background rate. Sample cuvettes contained 25 µL of supernatant combined with 2.0 mL of CS cocktail. Reactions were started by adding 25 µL of 0.2 mM oxaloacetate to sample cuvettes. The slopes of the background rates were subtracted from the slopes of the oxaloacetate-dependent rates to determine total CS activity. The calculated activity was reported as International Units (IU) per gram fresh tissue weight. Assays were initially run at both acclimations temperatures (−1°C and 4°C). Aside from Q_10_ effects on the overall rate of the reaction, no significant changes in the absolute differences between treatments was observed between assays run at −1°C and 4°C. Therefore, results reported below represent assays performed at −1°C for all samples.

#### Lactate dehydrogenase activity

We also used a spectrophotometric method to quantify total lactate dehydrogenase (LDH) activity from gill and liver tissues. Tissue extracts were prepared as described above, and run in duplicate with enzyme activity measured at −1°C. To determine the total LDH activity, 5 µL of cleared supernatant was combined with 2.00 mL LDH cocktail solution (0.20M Imidazole-HCl buffer, pH = 7.0, 5.50 mM NADH, 2.00 mM sodium pyruvate) and the change in absorbance at 340nm was measured over a 3 min period. The slope of the absorbance change was used to calculate the LDH activity rate and was reported as IU per gram fresh weight. As with CS activity assays, a Q_10_ effect was noted, but relative differences in enzyme activity between treatments did not differ based on assay temperature. All results reported represent assays run at −1°C.

### Statistical analysis

All experimental metrics except for RMR were analyzed using a 3-way ANOVA, with acclimation time, temperature, and *p*CO_2_ level used as main effects. As oxygen consumption rates for the same group of fish were measured at all four time points, we fit a multivariate model for repeated measures (MANOVA-RM) with RMR at 7, 28, 42 and 56 days of acclimation as dependent variables and temperature and *p*CO_2_ level as main effects to assess changes in RMR over time. For cases in which only main effects were found with no interaction, an uncorrected *t*-test was employed to elucidate significant differences between experimental treatments.

Several published recommendations have suggested that post-hoc corrections can lead to overly conservative analyses and loss of statistical power given the prior rejection of the omnibus null hypothesis (e.g. Rothman, 1990; Hurlbert and Lombardi, 2003; Nakagawa, 2004; Hurlbert and Lombardi, 2012). Specifically, use of post-hoc corrections can lead to the scientifically uninterpretable result that there are no differences between groups when the null hypothesis that the group means are all equal has already been rejected. For this reason, we follow significant omnibus effects tests for nominal variables with uncorrected *t*-tests. All statistical analyses were performed using the JMP^**®**^11 statistical software.

## Results

### Fulton's Index and growth rate

**Figure 1:**
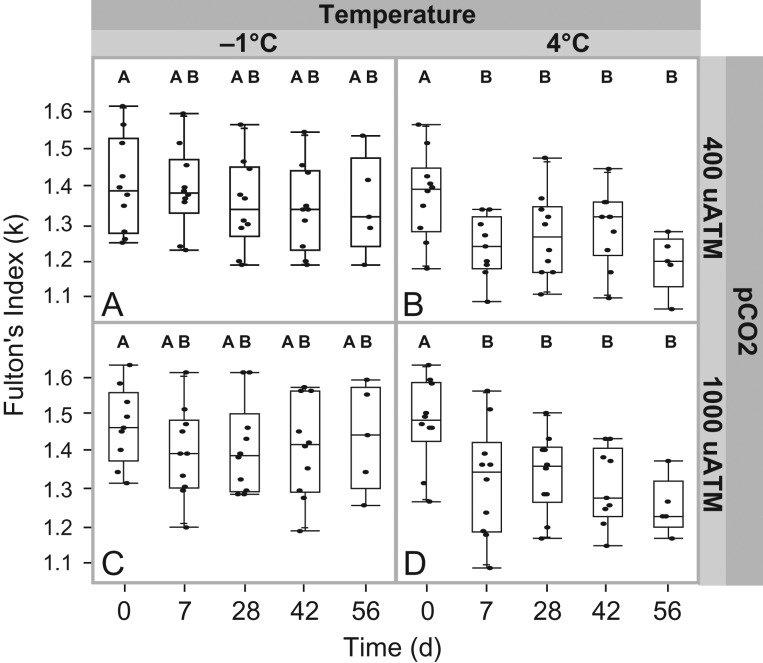
Fulton's Index (±SE) calculated at 0, 7, 28, 42 and 56 days in *Trematomus bernacchii* to a control treatment (**A**), high temperature + low *p*CO_2_ (**B**), low temperature + high *p*CO_2_ (**C**), and high temperature + high *p*CO_2_ (**D**). Time points within a treatment not connected by the same letter are significantly different from each other.

Overall, *T. bernacchii* was the only species that displayed any significant impact of the acclimation treatments on the mass of the fish over the course of the 56-day experiment. We found overall, that the calculated Fulton's condition index and growth rate significantly declined in *T. bernacchii* over the course of the 56-day acclimation period despite being fed to satiation (*F*_(19,158)_ = 4.601, *P* < 0.001; Fig. 1). We found a main effect of temperature (*F*_(1,158)_ = 9.816, *P* = 0.002) and time (*F*_(4,158)_ = 4.043, *P* = 0.003) on the condition index of *T. bernacchii,* with no interaction among the main effects. Analysis of growth rates also showed a significant decline (*F*_(15,140)_ = 2.1564, *P* < 0.011) with a main effect of temperature (*F*_(1,140)_ = 5.198, *P* < 0.024), with no apparent interaction between treatments and time (*P* = 0.447). Elevated temperature appears to be the major driver of the decline in fish condition and growth (Table 2; Fig. 1). The largest decline of growth and condition were seen in the multi-stress fish at 7 days of acclimation, followed by the high temperature fish at 7 days of acclimation. Overall, while both growth and condition declined over the course of the experiment, this decline slowed over time (Table 2; Fig. 1).

### Routine metabolic rates

**Figure 2:**
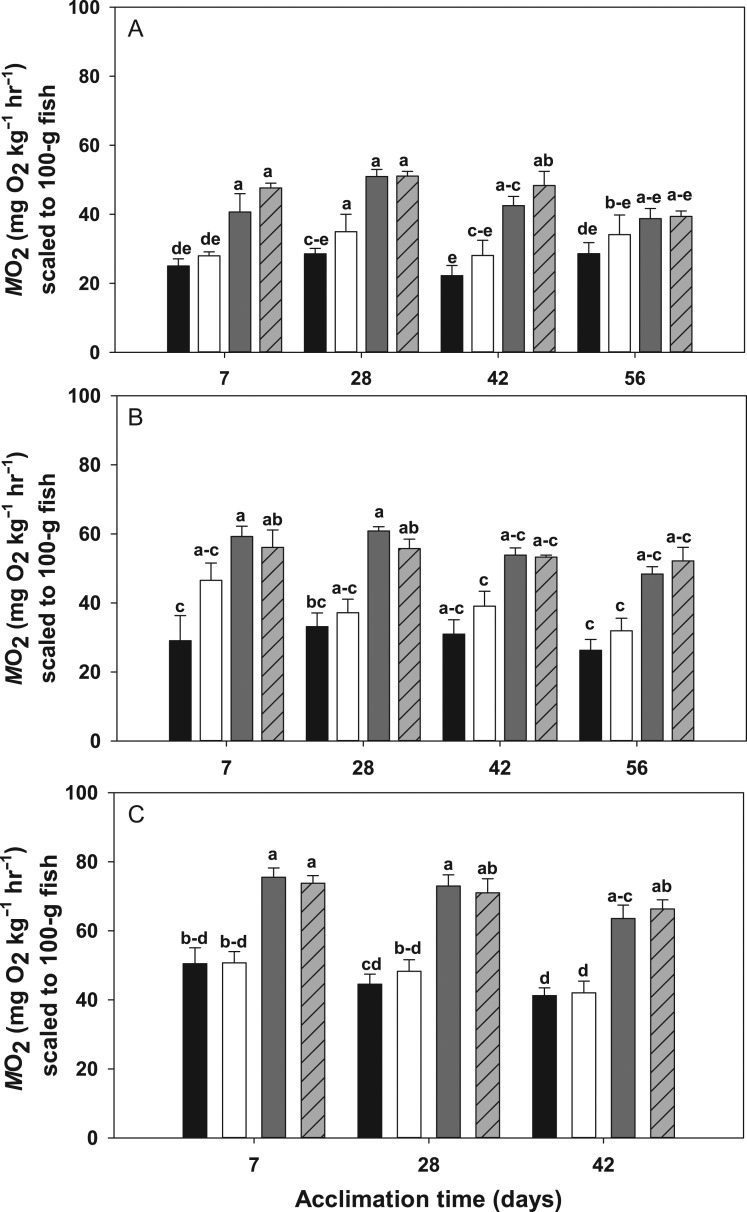
${\overset{˙}{M}O}_{2}$ values, scaled to 100-g fish (±SE) for *Trematomus bernacchii* (**A**), *Pagothenia borchgrevinki* (**B)**and *Trematomus newnesi* (**C**) acclimated at 7, 28 and 42 or 56 days to a control treatment (low temperature + low *p*CO_2_; black bars), low temperature + high *p*CO_2_ (white bars), high temperature + low *p*CO_2_ (dark gray bars) and high temperature + high *p*CO_2_ (light gray bars with crosshatches). Groups not connected by the same letter are significantly different from each other.

Oxygen consumption rates from *T. bernacchii* showed significant differences across treatment groups (*F*_(3,15)_ = 3.154, *P* < 0.001) with a significant main effect of temperature (*F*_(1,15)_ = 1.693, *P* < 0.001). Fitting of a multivariate model (MANOVA with repeated measures) revealed no significant interactions between the main effects (*P* = 0.152) as well as no significant effects of time within treatment groups (*P* = 0.186). Overall, temperature had a pronounced effect across time, significantly elevating RMR's in both high temperature treatments at 7, 28 and 42 days of acclimation (Fig. 2A, *P* < 0.001). In the 56-day acclimation group, RMR dropped in the high temperature acclimation group (4°C and 430 µatm), becoming only marginally significant (*P* = 0.052). A small elevation in RMR was noted at 28 days of acclimation in the high **P**CO_2_ treatment, yet returned to control levels at the 42-day time point (Fig. 2A).

For *P. borchgrevinki* we also found a significant effect of treatment (*F*_(3,15)_ = 3.056, *P* < 0.001) with a significant main effect of temperature (*F*_(1,15)_ = 1.750, *P* < 0.001) and no significant interactions (*P* = 0.259). While temperature had a significant effect on RMR's in *P. borchgrevinki* over shorter acclimation periods (7 and 28 days, *P* < 0.001), unlike *T. bernacchii,* oxygen consumption rates in both high temperature treatments declined over time and were indistinguishable from control fish by 42 days of acclimation (Fig. 2B).

Similar to both *T. bernacchii* and *P. borchgrevinki*, we again found a significant difference in RMR between acclimation groups (*F*_(3,15)_ = 6.910, *P* < 0.001) with a main effect of temperature (*F*_(1,15)_ = 3.814 *P* < 0.001) in *T. newnesi*. There was no significant interaction between temperature and *p*CO_2_ (*P* = 0.907), and time had no significant effect within treatment groups. (*P* = 0.246). As seen with both *T. bernacchii* and *P. borchgrevinki,* treatments involving elevated temperature resulted in a significant increase in oxygen consumption rates after 7 and 28 days of acclimation (Fig. 2C, *P* < 0.001). However, unlike *P. borchgrevinki*, the RMR of *T. newnesi* remained elevated even after 42 days of acclimation (Fig. 2C, *P* < 0.001). Unfortunately, given the time constraints of our field season, we were unable to obtain a measurement of RMR in *T. newnesi* beyond 42 days and it is unknown if their RMR in the high temperature + low *p*CO_2_ treatment returns to basal levels after 56 days of acclimation similar to *T. bernacchii*.

### Lipid analysis

Only small changes were noted in all three species with respect to the concentration of total triglycerides in liver tissues over time (Table 2, supplemental data, S1 & S2). We observed no significant effect of temperature, *p*CO_2_, or acclimation time in any species (Table 2, supplemental data, S1 & S2). Similar results were observed in white muscle, another major lipid storage site in these fishes (Table 2, supplemental data, S1 & S2).

### Citrate synthase activity

**Figure 3:**
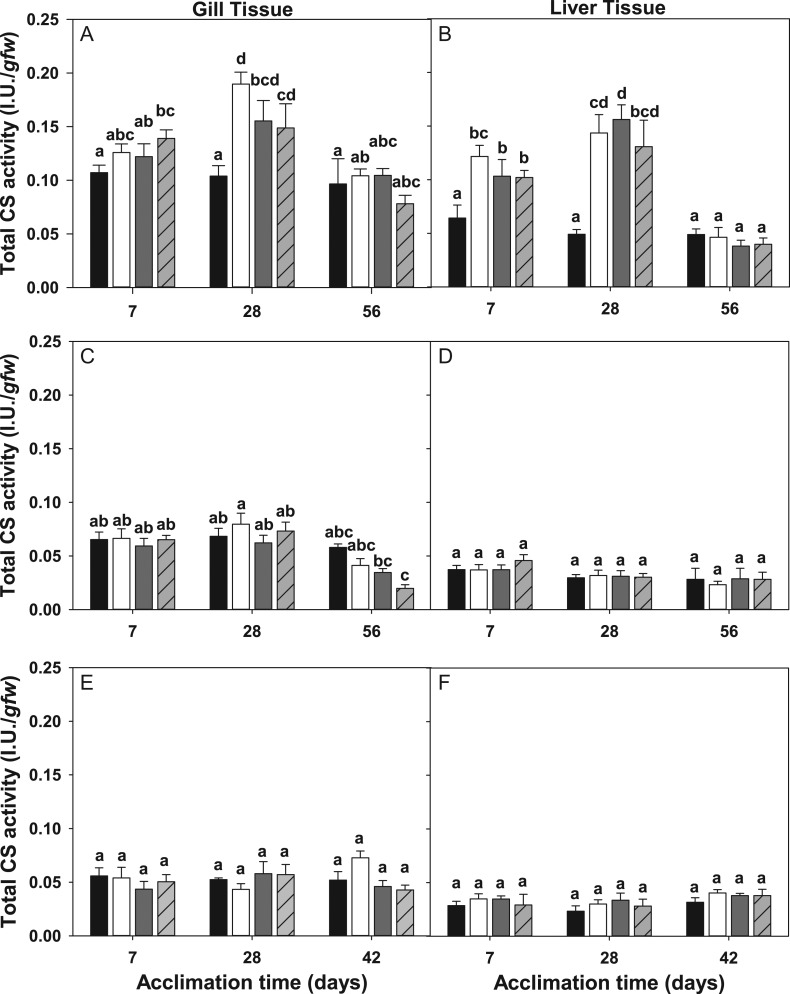
Citrate synthase enzyme activity (±SE) of *Trematomus bernacchii* gill (**A**) and liver tissues (**B**), *Pagothenia borchgrevinki* gill (**C**) and liver tissues (**D**) and *Trematomus newnesi* gill (**E**) and liver tissues (**F**) acclimated at 7, 28 and 42 or 56 days to a control treatment (low temperature + low *p*CO_2_; black bars), low temperature + high *p*CO_2_ (white bars), high temperature + low *p*CO_2_ (dark gray bars) and high temperature + high *p*CO_2_ (light gray bars with crosshatches). Groups not connected by the same letter are significantly different from each other.

We observed unique patterns of changes in the total CS activity across all three species. Both gill and liver tissues from *T. bernacchii* displayed significant differences in CS activity between acclimation treatments and tissues (*F*_(11,80)_ = 2.138, *P* = 0.035 (gill), *F*_(11,80)_ = 2.544, *P* = 0.016 (liver)). In gill tissues, a significant increase in activity occurred within the first 7 days of acclimation in the multi-stress treatment and persisted through the 28-day acclimation time point in all treatments compared to control fish (Fig. 3A,B). In liver tissues, CS activities significantly increased in all treatments at both the 7 and 28-day time points. A significant effect of time was found in both tissues (*F*_(2,80)_ = 4.517, *P* < 0.001 (gill)), (*F*_(2,80)_ = 3.377, *P* = 0.002 (liver)), highlighted by an initial increase in enzyme activity followed by a large drop in CS activity at 56 days (Fig. 3A,B). Both tissues also showed a main effect of *p*CO_2_ (*F*_(1,80)_ = 4.578, *P* = 0.040 (gill)), (*F*_(1,80)_ = 10.12, *P* = 0.003 (liver)), but temperature was only found to have a significant effect in liver tissues (*F*_(1,80)_ = 6.03, *P* = 0.02). We also observed a significant interaction between time, temperature and *p*CO_2_ level in liver tissues isolated from *T. bernacchii* (*F*_(4,80)_ = 7.473, *P* = 0.002), and this interaction appears to be largely antagonistic in nature, illustrated by the decreased activity level in the multi-stress treatment compared to the single stress treatments (Fig. 3B, supplemental data, S4).

In *P. borchgrevinki* there appeared to be a tissue-specific response with respect to total CS activity. We found a significant difference in CS activity between treatments within gill tissues (*F*_(11,80)_ = 4.47, *P* < 0.001) with a main effect of time (*F*_(2,80)_ = 15.789, *P* < 0.001) and temperature (*F*_(1,80)_ = 5.663, *P* = 0.02). Furthermore, unlike the patterns observed in *T. bernacchii,* CS activity in gill tissues isolated from *P. borchgrevinki* showed relatively small changes in CS activity and no significant differences were observed between treatments in either tissue (Figs. 3C,D).

Of all three species, CS activity in *T. newnesi* displayed the least sensitivity to the acclimation treatments. While gill and liver tissues showed small increases in activity relative to control values, these changes were not statistically significant. (Fig. 3E, F).

### Lactate dehydrogenase activity

**Figure 4: cox019F4:**
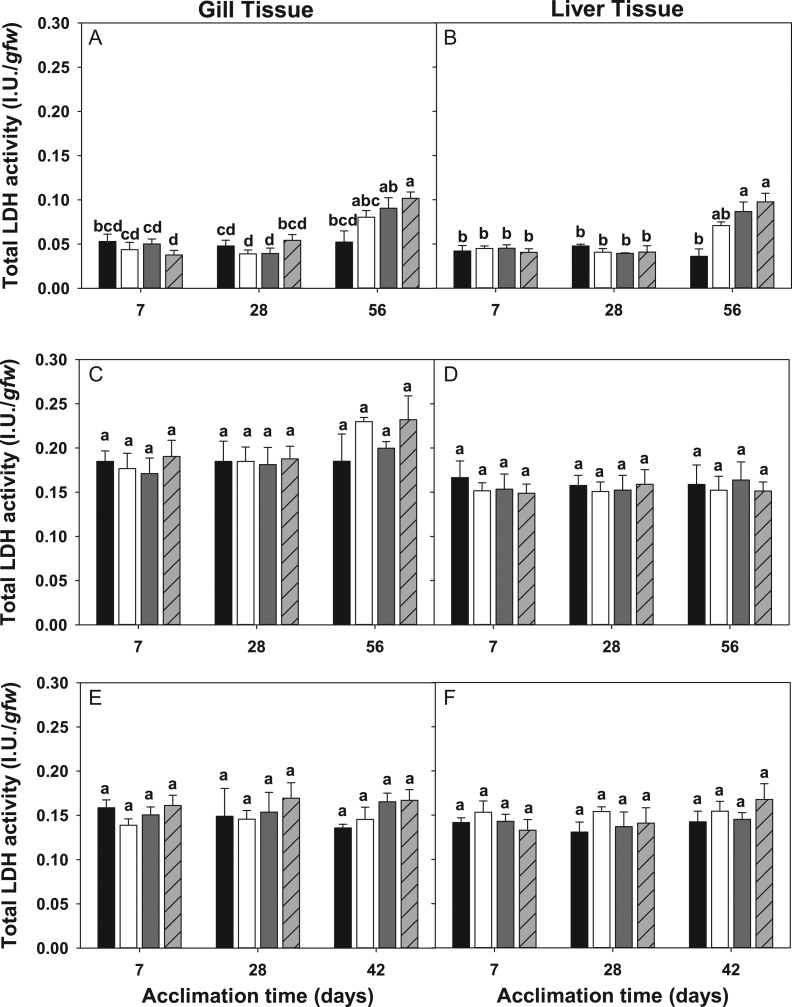
Lactate dehydrogenase enzyme activity (±SE) of *Trematomus bernacchii* gill (**A**) and liver tissues (**B**), *Pagothenia borchgrevinki* gill (**C**) and liver tissues (**D**) and *Trematomus newnesi* gill (**E**) and liver tissues (**F**) acclimated at 7, 28 and 42 or 56 days to a control treatment (low temperature + low *p*CO_2_; black bars), low temperature + high *p*CO_2_ (white bars), high temperature + low *p*CO_2_ (dark gray bars) and high temperature + high *p*CO_2_ (light gray bars with crosshatches). Groups not connected by the same letter are significantly different from each other.

*Trematomus bernacchii* was the only species in this study that displayed a significant change in LDH activity (*F*_(11,80)_ = 7.59, *P* < 0.001, (gill), *F*_(11,80)_ = 8.94, *P* < 0.001 (liver)). In *T. bernacchii*, both gill and liver tissues showed a main effect of time on LDH activities (*F*_(2,80)_ = 26.29, *P* < 0.001 (gill), *F*_(2,80)_ = 29.606, *P* < 0.001 (liver)) while liver tissues also showed a main effect of temperature (*F*_(1,80)_ = 6.192, *P* = 0.017). In gill and liver tissues, LDH activity in fish acclimated to experimental treatments were indistinguishable from control fish until 56 days of acclimation, at which point LDH activity increased significantly (Fig. 4A, [Fig cox019F4]B).

Unlike the robust response after 56 days of acclimation in *T. bernacchii, P. borchgrevinki* and *T. newnesi* displayed little to no change in LDH activity in either gill or liver tissue (Fig. 4C–F).

## Discussion

Given the recent establishment of the first ever marine protected area in the Ross Sea, a clearer understanding of the susceptibility of fish populations in these waters is critical to identifying how to better approach management of this unique marine ecosystem. As such, findings from comparative studies that address the capacity for endemic fish of the Southern Ocean to offset environmental changes will help inform efforts to predict population level responses to global climate change and forecast the sensitivity of this unique ecosystem. Notothenioid fishes occupy critical positions in the food web of the Ross Sea, serving as critical links between the lower trophic levels and higher trophic levels occupied by top predators (see La Mesa *et al*., 2004 for a review). These fishes occupy nearly all of the available trophic niches, serving as important predators of benthic invertebrates, zooplankton and other fish. They also serve as important food sources for many of the birds and mammals that occupy the upper levels of the food web. In particular, *T. bernacchii, T. newnesi* and*P. borchgrevinki* constitute important prey species consumed by top predators such as Weddell seals, emperor and Adélie penguins, as well as the south polar skua (Castellini *et al*., 1992; Mund and Miller, 1995; Burns *et al*., 1998; Clarke *et al*., 1998; Ainley *et al*., 2002; Polito *et al*., 2002). Therefore, comparative studies such as this may play an important role in future attempts to create a framework for predicting population level responses for the Notothenioidei suborder as a whole.

Acclimation to hypercapnia or elevated temperature elicits a suite of physiological changes (e.g. decreases in intracellular/extracellular pH, Heisler, 1984) that result in at least short-term disturbances in the energy balance of fish (Hochachka and Somero, 2002). Changes in RMR can provide early indicators of this energetic imbalance, and when tracked over long acclimation periods, can provide some insight into the capacity of fish to compensate for intracellular disturbances. In general, the measurement of whole animal metabolic rates provides an initial indicator of a species’ thermal tolerance, as limits in oxygen consumption can reflect the onset of whole animal oxygen limitation and associated limitations in circulatory capacity (Pörtner and Knust, 2007; Pörtner, 2010). Nonetheless, an in-depth understanding of the impact increased temperature and *p*CO_2_ have on the bioenergetics of an organism benefits from the study of cellular level indices of metabolic capacity (Sokolova *et al*., 2012; Sokolova, 2013; Todgham and Stillman, 2013), and therefore, should be examined along with whole-organism observations when feasible. Using both whole-organism measurements and enzymatic analysis of aerobic and anaerobic pathways, we have highlighted distinct physiological responses elicited by ocean acidification and increased SSTs among three closely related notothenioid species.

Our data show that while elevated *p*CO_2_ alone had little long-term impact on metabolic demands in these fish, elevation in temperature showed profound and lasting impacts on the energetic demands on two of the three notothenioid species studied. Although it appears the capacity to acclimate to increases in temperature and/or *p*CO_2_ are present in the Antarctic fish in this study, this capacity may be limited in *T. bernacchii* and *T. newnesi*. These patterns follow similar trends that were previously noted in these species after 28 days of acclimation to the same treatment levels despite employing different experimental approaches (Enzor *et al*., 2013).

Previous work on *P. borchgrevinki* has shown that these fish are capable of acclimating to 4°C after ~ 4–6 weeks, suggesting a capacity to re-establish energetic balance after exposure to a single chronic stress (Seebacher *et al*., 2005; Franklin *et al*., 2007; Robinson and Davison, 2008a, b; Bilyk and DeVries, 2011; Bilyk *et al*., 2012; Enzor *et al*., 2013). When given a chronic exposure to multiple stressors, *P. borchgrevinki* displayed a similar capacity to acclimate. After 42 days of acclimation, *P. borchgrevinki* RMR's were no longer significantly elevated above control values, suggesting complete compensation to both elevated temperature and *p*CO_2_. When acclimated to elevated temperatures alone, we found *T. bernacchii* also displays complete compensation at 4°C which concurs with previous results reported by Sandersfeld *et al*. (2015). Quite notably, however, both our data and the results reported by Sandersfeld *et al*. (2015) suggest the time-frame for complete acclimation is considerably extended in *T. bernacchii*, requiring somewhere between 8 and 9 weeks. Furthermore, unlike *P. borchgrevinki*, the combination of multiple stressors results in only partial compensation in *T. bernacchii* and *T. newnesi* as RMR's of these species remained elevated above control throughout the duration of the experiment. This partial compensation may highlight the beginning of limitations in oxygen delivery to metabolically active tissues.

Pörtner and colleagues have previously postulated that limitations of the cardiovascular system in Antarctic fish may lead to a reduced cardiac scope at elevated temperatures and inefficiency in oxygen delivery (Mark *et al*., 2002; Pörtner and Knust, 2007; Pörtner, 2010; Strobel *et al*., 2012), which in turn, may impact the way temperature affects specific dynamic action in fish (Fry, 1971; Jobling, 1981). Strobel *et al*. have previously linked warm acclimation to reduced fish condition in the Antarctic teleost *Notothenia rossi* despite being fed *ad libitum* (2012). We found temperature also played a significant role in the condition factor and growth of *T. bernacchii*. Values for Fulton's condition index (*K*) as well as growth rates decreased in warm acclimated specimens of this species over time despite being fed to satiation. This decrease in fish condition suggests that *T. bernacchii* may not be capable of ingesting sufficient food over time to meet the required energy demand and may experience a decrease in scope for growth. These results correspond with the findings of Sandersfeld and colleagues who observed a significant reduction in body mass for *T. bernacchii* when exposed to elevated temperature alone (Sandersfeld *et al*. 2015). While a significant effect of treatment was identified, it should also be noted that control fish showed little to no growth over the course of this experiment despite being feed *ad libitum.* This may signal that the overall energetic status of the fish in captivity is less than optimal even under control conditions. It is unclear if the choice of food resulted in a reduced assimilation efficiency, which could be further compounded by a reduced assimilation rate induced by elevated temperatures as suggested by Sandersfeld *et al*. (2015). Sandersfeld further noted refusal of food despite being offered unlimited amounts. Therefore, despite being fed *ad libitum* the fish in our study may not have actually been satiated. Given the small sample size and lack of growth in our control samples, the long-term implications of these stressors on the growth of these fish should be interpreted with caution.

To maintain elevated RMR's in warmer waters, energy stores such as lipid reserves may be mobilized which would lead to a further reduction in fish condition. When acclimated to elevated temperature alone, an increase in the expression of apolipoproteins associated with lipid transport has been previously observed in *T. bernacchii* (Huth and Place, 2013), however, when *T. bernacchii* was acclimated to the combined stress of elevated temperature and *p*CO_2_, lipid mobilization appeared to be significantly down-regulated with sixteen genes involved in these pathways (i.e. lipoprotein lipase, fatty acid hydrolase and lipocalin) displaying a 2-fold or greater decrease in expression (Huth and Place, 2016b). Our biochemical analysis of lipid content in *T. bernacchii* supports the transcript level data previously published by Huth and Place (2016b) in that we did not observe a change in lipid concentration despite the significant decline in body mass, suggesting mobilization of lipids has at least slowed in these fish. As an alternative to mobilizing energy stores, fish may be employing physiological trade-offs or shifting energetic pathways in an effort to reduce oxygen demand at the tissue level and protect metabolic scope and scope for growth (Michaelidis *et al*., 2007; Windisch *et al*., 2011; Mogensen and Post, 2012). Interestingly, *P. borchgrevinki*, which displayed relatively few metabolic changes in this study, did not display a comparable change in expression of genes involved in lipid mobilization after acclimation to elevated temperatures (Bilyk and Cheng, 2014; Huth and Place, 2016a) suggesting *P. borchgrevinki* requires little physiological adjustment to acclimate to elevated temperatures. These data further support our assessment that *P. borchgrevinki* may be more tolerant of environmental perturbation than closely related benthic notothenioids (Seebacher *et al*., 2005).

Analysis of metabolic enzyme capacity in these three species after acclimation to a dual-stressor treatment provides further insight into the different physiological responses of these closely related notothenioids. While both *T. newnesi* and *T. bernacchii* show signs of a reduced capacity to metabolically compensate after warm acclimation, biochemical analysis of aerobic and anaerobic metabolism suggest they may be utilizing different approaches to reduce oxygen demand at the cellular level. Although variable across treatments and time points, *T. newnesi* showed no significant changes in either CS or LDH capacity. The lack of compensation in glycolytic capacity suggests *T. newnesi* is perhaps relying on physiological trade-offs to deal with the energetic imbalance induced by elevated temperature and *p*CO_2_. Unlike what we observed in *T. newnesi* and *P. borchgrevinki*, specimens of *T. bernacchii* showed a significant change in glycolytic capacity after acclimation to elevated *p*CO_2_ and temperature, a response also observed in single stressor studies in other notothenioid species (Strobel *et al*., 2012, 2013a). By extending the acclimation time, we were able to observe the initial increase in CS activity was followed by a swift decline, coupled with a significant increase in LDH activity at 56 days. This initial increase in glycolytic capacity, followed by a rise in LDH, may signal a heavier dependence of ATP-generation via glycolysis. Energy for gill cell function is primarily supplied by the oxidation of glucose and lactate obtained from the circulation as a result of carbohydrate metabolism in most teleost fishes (Perry and Walsh, 1989; Morgan *et al*., 1997). Furthermore, the Cori cycle is thought to have a negligible role in teleost fishes making circulating lactate available for use by other organ systems (see Milligan 1996 for review). Given the increases in LDH observed in the gill we suspect the gill cells are capable of converting the available lactate in the blood back to pyruvate, supplementing the pyruvate produced through glycolysis. This could also help explain why we see changes in condition factor coupled with decline in growth rates, but no changes in lipid content. These findings are also reflected at the level of the transcript in *T. bernacchii* as well. Huth and Place (2016b) found induction of multiple genes involved in carbohydrate metabolism that were accompanied by down-regulation of lipid-mobilization and beta-oxidation pathways which further suggests a heavier reliance on glycolytic substrates in these fish when acclimated to the dual-stressor. Jayasundara and colleagues (2013) previously noted a similar tissue-specific increase in LDH activity following acclimation to warm temperature, which was attributed to circulatory limitation. While oxygen limitation may indeed be at play here, it is difficult to reconcile this explanation with the rapid increase in CS also noted in our study. Alternatively, this possible switch in substrate usage may be driven by a significant increase in oxidative damage induced by the elevation of metabolic rates in these fish (Enzor and Place, 2014). We have previously postulated *T. bernacchii* might employ the use of alternative energy sources as a means to combat this oxidative damage. Although we have yet to test this empirically, there are several lines of evidence to suggest this is plausible. The increase in LDH and decrease in CS is mirrored by a significant drop in oxidatively damaged proteins in *T. bernacchii* (Enzor and Place, 2014). The β-oxidation of lipids can be a substantial source of reactive oxygen species (Abele and Puntarulo, 2004), thus switching to carbohydrate metabolism may help offset the effects of increased RMR. Furthermore, as reactive oxygen species are primarily formed in the third mitochondrial complex of the electron transport chain (Murphy, 2009), supplementing energy production with anaerobic pathways may act to further decrease reactive oxygen species formation and subsequent oxidative damage. Lastly, *T. newnesi* did not show similar changes in glycolytic capacity and also displayed higher levels of oxidative damage when acclimated to elevated temperature and *p*CO_2_ (Enzor and Place, 2014), which lends further support to the mechanistic link between substrate switching and oxidative damage in *T. bernacchii*.

### Conclusions

In an era of research devoted to global climate change, one of the main questions put forth by scientists is whether or not species can acclimate and in turn adapt to predicted changes in our global oceans. In this study, we attempted to discern the acclimation capacity of three species of notothenioid fish to simultaneous increases in both SST and seawater *p*CO_2_ levels. Our data suggest that although most notothenioid species have evolved in the same stable, cold environment for millions of years, these fishes are unlikely to physiologically respond to climate change stressors in the same manner. Similar to findings from previous dual-stressor studies, it appears the partial compensation of RMRs observed in *T. bernacchii and T. newnesi* are induced by temperature alone, and elevated *p*CO_2_ has little impact on this compensation in any of the species studied here (Strobel *et al*., 2012, 2013a, b; Enzor *et al*., 2013; Enzor and Place, 2014). Furthermore, examination of cellular-level processes along with organismal condition suggests energetic trade-offs may underlie the acclimation response of at least one of the three species. Lastly, although all three species displayed at least partial compensation for the intracellular changes induced by elevated temperature, the inability of some notothenioid species to fully compensate for the energetic demands of chronic exposure to elevated temperature suggests there is the potential for population level impacts on growth and reproduction to manifest in a number of fishes inhabiting the Ross Sea.

## Supplementary Material

Supplementary Data

Supplementary Data

Supplementary Data

Supplementary Data
