# Supplementary figures and images for: The effects of elevated temperature and ocean acidification on the metabolic pathways of notothenioid fish
Source: Conserv Physiol. 2017 Mar 24;5(1):cox019. doi: 10.1093/conphys/cox019 (PMC5570038; doi:10.1093/conphys/cox019)

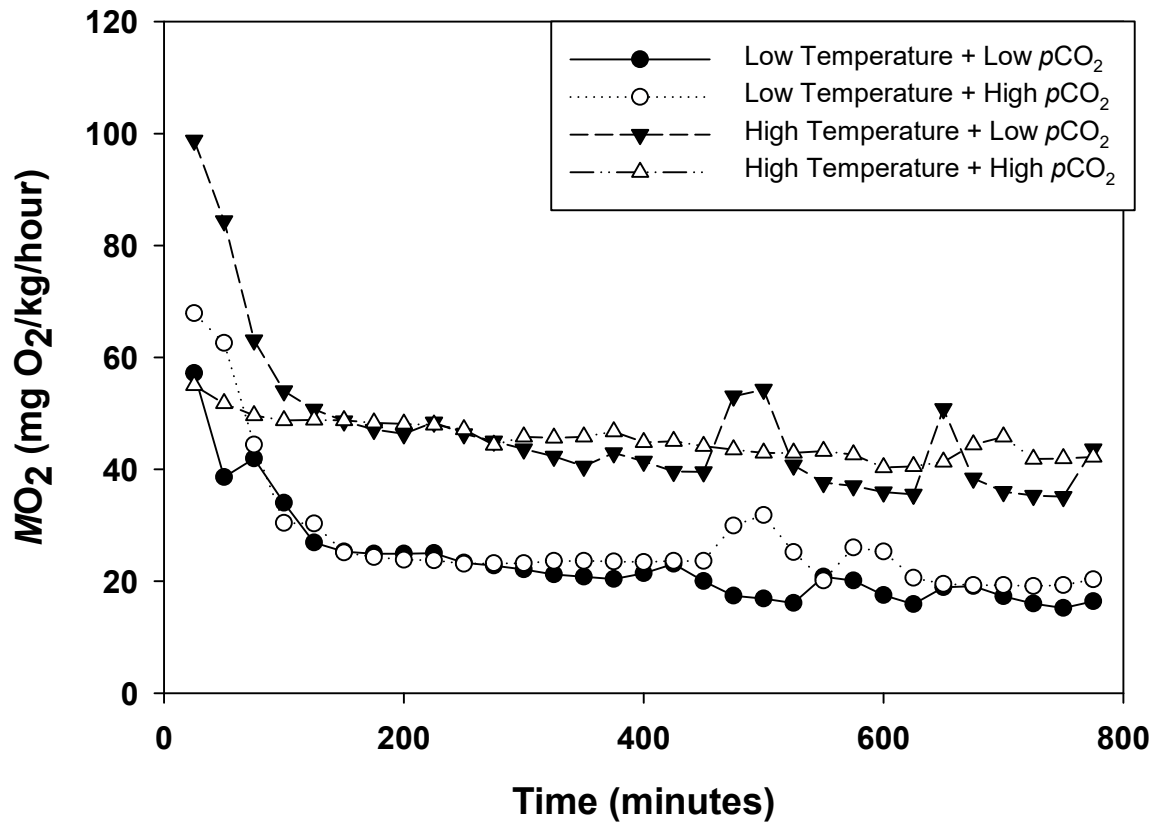

Supplement: Supplementary Data [file S3LoligoTrace.pdf]

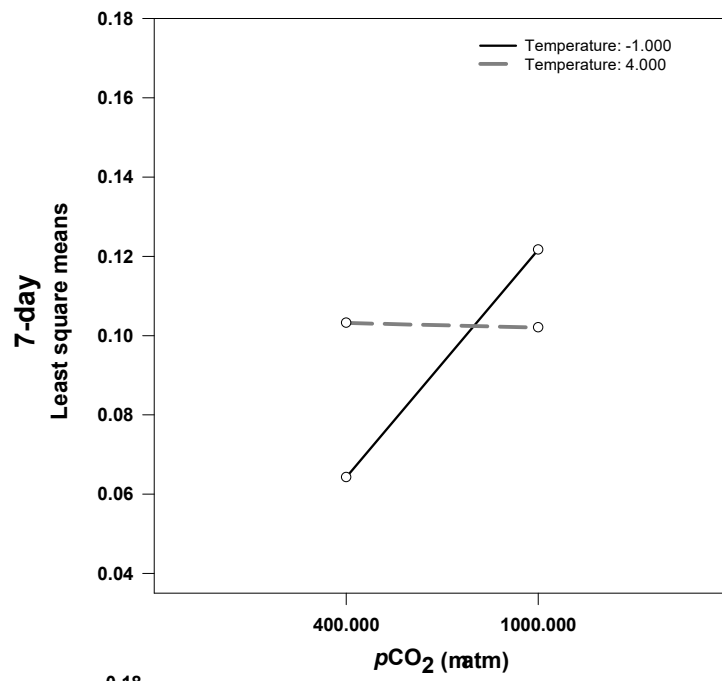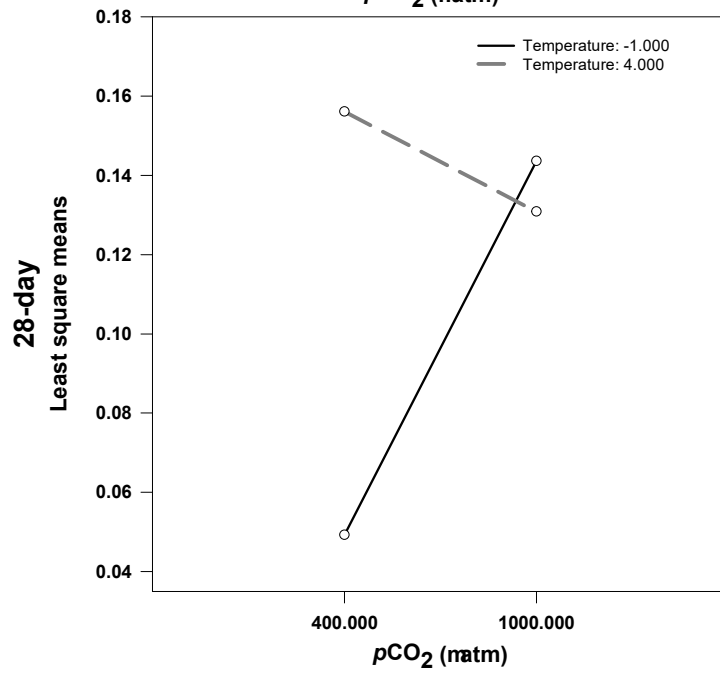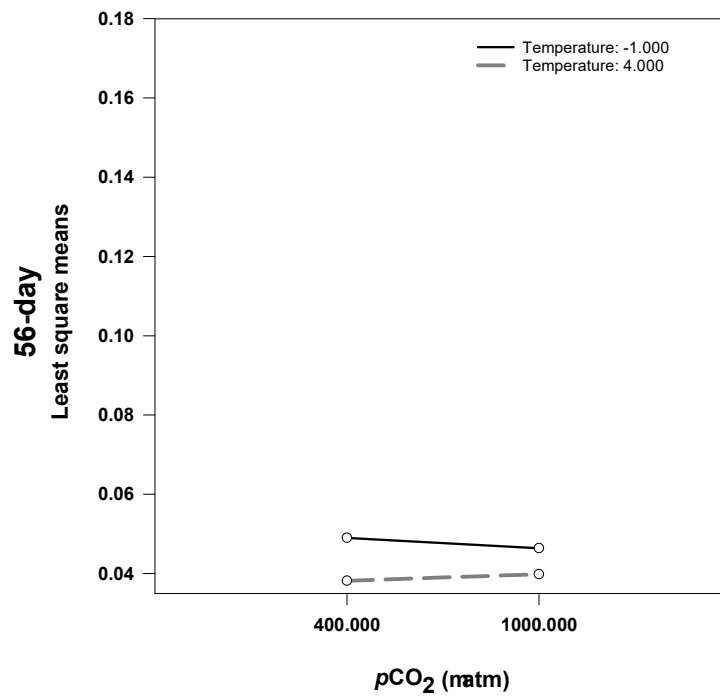

Supplement: Supplementary Data [file S4CSInteractions2-20-17.pdf]
