# Supplementary material for: The effects of elevated temperature and ocean acidification on the metabolic pathways of notothenioid fish
Source: Conserv Physiol. 2017 Mar 24;5(1):cox019. doi: 10.1093/conphys/cox019 (PMC5570038; doi:10.1093/conphys/cox019)
Supplement: Supplementary Data [file TableS1Pb.docx]

Supplementary Table S1: Fish condition, growth parameters and lipid concentrations for *Pagothenia borchgrevinki*

|  | Acclimation time (d) | Low Temp + Low *p*CO_2_ | Low Temp + High *p*CO_2_ | High Temp + Low *p*CO_2_ | High Temp + High *p*CO_2_ |
| --- | --- | --- | --- | --- | --- |
| Mortality  (sample size) | 7 |  |  |  |  |
|  | 28 |  |  |  | 1 (day 12)*  (n=9) |
|  | 42 |  | 1 (day 28)*  (n=9) | 1 (day 5)*  (n=9) | 1 (day 11)*  (n=9) |
|  | 56 | 1 (day) 44* (n=4) |  |  |  |
| k | T0 | 0.979± 0.05 | 0.931± 0.02 | 0.965± 0.03 | 0.945± 0.05 |
|  | 7 | 0.914± 0.01 | 0.921± 0.02 | 0.912± 0.02 | 0.967± 0.03 |
|  | 28 | 0.960± 0.04 | 0.915± 0.03 | 0.954± 0.04 | 0.955± 0.02 |
|  | 42 | 0.950± 0.04 | 0.926± 0.03 | 0.905± 0.03 | 0.923± 0.02 |
|  | 56 | 0.902± 0.01 | 0.867± 0.02 | 0.873± 0.01 | 0.927± 0.03 |
| SGR  (% M day ^-1^) | 7 | -0.015± 0.02 | -0.021± 0.03 | -0.025± 0.02 | -0.025± 0.03 |
|  | 28 | 0.010± 0.03 | 0.018± 0.06 | -0.066± 0.04 | -0.028± 0.04 |
|  | 42 | 0.037± 0.05 | 0.004± 0.05 | -0.010± 0.05 | -0.037± 0.03 |
|  | 56 | 0.100± 0.07 | 0.003± 0.04 | -0.035± 0.04 | -0.098± 0.03 |
| Liver Lipids  (*n*=9) | 7 | 6.132± 0.60 | 6.692± 0.62 | 6.087± 0.60 | 6.666± 0.79 |
|  | 28 | 7.341± 0.67 | 7.639± 0.87 | 6.173± 0.33 | 7.563± 0.84 |
|  | 56 | 6.088± 1.47 | 6.012± 0.63 | 6.755± 0.86 | 6.818± 1.51 |
| WM Lipids  (*n*=9) | 7 | 1.752± 0.16 | 2.282± 0.33 | 1.739± 0.14 | 2.535± 0.30 |
|  | 28 | 2.469± 0.39 | 2.288± 0.24 | 1.763± 0.19 | 2.161± 0.20 |
|  | 56 | 1.740± 0.33 | 1.718± 0.16 | 1.930± 0.22 | 1.718± 0.45 |

Data are means ± SE; number of fish (n)=10 for all treatments except 56d time points (n=5) unless otherwise stated. Fulton’s condition index (k). Specific Growth Rate (SGR, % change in mass (M) per day ±SE). Lipid content (total triglycerides gfw^-1^, ±SE) of liver and white muscle (WM) of *Pagothenia borchgrevinki* at each experimental time point. *All mortality events were associated with fish that displayed early signs of x-cell disease after close examination post-acclimation period.
