# Supplementary material for: The effects of elevated temperature and ocean acidification on the metabolic pathways of notothenioid fish
Source: Conserv Physiol. 2017 Mar 24;5(1):cox019. doi: 10.1093/conphys/cox019 (PMC5570038; doi:10.1093/conphys/cox019)
Supplement: Supplementary Data [file TableS2Tn.docx]

Supplementary Table S2: Fish condition, growth parameters and lipid concentrations for *Trematomus newnesi*

|  | Acclimation time (d) | Low Temp + Low *p*CO_2_ | Low Temp + High *p*CO_2_ | High Temp + Low *p*CO_2_ | High Temp + High *p*CO_2_ |
| --- | --- | --- | --- | --- | --- |
| Mortality  (sample size) | 7 |  |  |  |  |
|  | 28 |  |  |  | 1 (day 10)  (n=9) |
|  | 42 |  |  |  |  |
| k | T0 | 1.209± 0.04 | 1.287± 0.06 | 1.325± 0.03 | 1.324± 0.05 |
|  | 7 | 1.222± 0.05 | 1.185± 0.04 | 1.217± 0.03 | 1.259± 0.04 |
|  | 28 | 1.246± 0.08 | 1.147± 0.04 | 1.203± 0.02 | 1.185± 0.03 |
|  | 42 | 1.298± 0.06 | 1.069± 0.03 | 1.183± 0.04 | 1.080± 0.01 |
| SGR  (% M day ^-1^) | 7 | -0.080± 0.02 | -0.152± 0.08 | -0.120± 0.07 | -0.187± 0.19 |
|  | 28 | 0.070± 0.04 | -0.092± 0.02 | -0.218± 0.04 | -0.100± 0.12 |
|  | 42 | 0.001± 0.02 | -0.084± 0.08 | -0.217± 0.03 | -0.223± 0.02 |
| Liver Lipids  (*n*=9) | 7 | 7.523± 1.16 | 6.608± 1.48 | 8.026± 1.65 | 8.219± 0.95 |
|  | 28 | 6.709± 1.34 | 7.324± 1.69 | 7.769± 2.19 | 7.213± 1.76 |
|  | 42 | 5.636± 0.57 | 6.185± 0.83 | 5.603± 1.37 | 6.593± 1.09 |
| WM Lipids  (*n*=9) | 7 | 1.413± 0.29 | 1.177± 0.25 | 1.840± 0.56 | 1.635± 0.35 |
|  | 28 | 1.165± 0.18 | 1.274± 0.26 | 1.351± 0.34 | 1.254± 0.27 |
|  | 42 | 0.978± 0.10 | 1.076± 0.14 | 0.974± 0.24 | 1.147± 0.17 |

Data are means ± SE; number of fish (n)=10 for all treatments unless otherwise stated. Fulton’s condition index (k). Specific Growth Rate (SGR, % change in mass (M) per day ±SE). Lipid content (total triglycerides gfw^-1^, ±SE) of liver and white muscle (WM) of *Trematomus newnesi* at each experimental time point.
